# AQP3 Promotes the Invasion and Metastasis in Cervical Cancer by Regulating NOX4-derived H_2_O_2_ Activation of Syk/PI3K/Akt Signaling Axis

**DOI:** 10.7150/jca.91360

**Published:** 2024-01-01

**Authors:** Qixin Wang, Bingjie Lin, Hongjian Wei, Xin Wang, Xiaojing Nie, Yonghua Shi

**Affiliations:** 1 Department of Pathology, School of Basic Medical Sciences, Xinjiang Medical University, Urumqi, Xinjiang 830017, China.; 2 Xinjiang Key Laboratory of Molecular Biology for Endemic Diseases, Xinjiang Medical University, Urumqi, Xinjiang 830017, China.

**Keywords:** AQP3, H_2_O_2_, Syk, Akt, Cervical cancer

## Abstract

Unrestrained chronic inflammation leads to the abnormal activity of NOX4 and the subsequent production of excessive hydrogen peroxide (H_2_O_2_). Excessive H_2_O_2_ signaling triggered by prolonged inflammation is thought to be one of the important reasons for the progression of some types of cancer including cervical cancer. Aquaporin 3 (AQP3) is a member of the water channel protein family, and it remains unknown whether AQP3 can regulate the transmembrane transport of nicotinamide adenine dinucleotide phosphate (NADPH) oxidase 4 (NOX4)-derived H_2_O_2_ induced by the stimulation of inflammatory factors to facilitate the malignant progression in cervical cancer. In this study, cervical cancer HeLa cell line was respectively treated with diphenyleneiodonium (DPI), N-Acetylcysteine (NAC) or lentivirus-shRNA- AQP3. Plate cloning, cell migration or transwell invasion assays, etc. were performed to detect the invasive and migration ability of the cells. Western blot and CO-IP were used to analyze the mechanism of AQP3 regulating H_2_O_2_ conduction. Finally, in vivo assays were performed for validation in nude mice. AQP3 Knockdown, DPI or NAC treatments all reduced intracellular H_2_O_2_ influx, and the activation of Syk/PI3K/Akt signal axis was inhibited, the migration and invasive ability of the cells was attenuated. *In vivo* assays confirmed that the excessive H_2_O_2_ transport through AQP3 enhanced the infiltration and metastasis of cervical cancer. These results suggest that AQP3 activates H_2_O_2_/Syk/PI3K/Akt signaling axis through regulating NOX4-derived H_2_O_2_ transport to contribute to the progression of cervical cancer, and AQP3 may be a potential target for the clinical treatment of advanced cervical cancer.

## 1. Introduction

Cervical cancer is the fourth leading cause of cancer death in women 1, 2. Uncontrolled chronic inflammation is frequently a significant and common factor contributing to cancer development and metastasis. Additionally, tissues affected by chronic inflammation often display disrupted microenvironments 3. Tumor microenvironment (TME) is a complex and highly heterogeneous dynamic comprehensive system, which is mainly composed of tumor cells, tumor-associated fibroblasts (CAFs), tumor-associated immune cells, and micro-vessels 4, 5. These cells are highly plastic and can continuously change their phenotype and function, forming an inflammatory microenvironment conducive to tumor development through direct cell-cell contact or dynamic crosstalk between soluble factors such as cytokines, chemokines, and growth factors 6. It is well known that persistent human papilloma-virus (HPV) infection is closely related to cervical cancer and, consequently, inflammation plays a key role in the occurrence and development of cervical cancer 7, 8.

A variety of inflammatory mediators in the inflammatory microenvironment, such as growth factors, cytokines, and hormones, usually bind to nicotinamide adenine dinucleotide phosphate oxidase (NOX) family on the membrane of cancer cells to catalyze the generation of reactive oxygen species (ROS) which are important developmental and physiological stimuli. NOX4 is predominantly detected in human tumor cell lines and has been linked to various cellular processes, including the formation of invadopodia 9, cell proliferation 10, differentiation 11, and epithelial-mesenchymal transition (EMT) 10. Transforming growth factor-β1 (TGF-β1) is one of the mediators in the inflammatory microenvironment of tumors, which is mainly manifested as a promoter during cancer progression. More importantly, cancer cells can utilize TGF-β signaling to induce EMT 12. The elevated ROS is found in pathological conditions such as cancer and is a major mediator contributing to the progression of cancers 13, 14. Hydrogen peroxide (H_2_O_2_) is the most abundant and stable ROS in living cells, low concentration of which was confirmed as a second messenger and played an important role in the biological processes under physiological conditions and cancer progress 15-17. However, the passive diffusion of extracellular H_2_O_2_ through the cell membrane is restricted 18. For efficient H_2_O_2_ signal transduction, there is a need for high-capacity and effective H_2_O_2_ transmembrane influx, underscoring the importance of regulating cell permeability.

Aquaporin 3 (AQP3) is a peroxiporin, in addition to transporting water and glycerol, which has been reported to promote cancer cell migration and invasion by transporting H_2_O_2_ 19, 20. H_2_O_2_, as a signaling molecule, promotes abnormal cell growth, metastasis, and angiogenesis 21, 22 via the activation of pro-survival signaling pathways, loss of tumor suppressor gene function, increase of glucose metabolism, adaptation to hypoxia, and the generation of carcinogenic mutations 22, 23. Inhibition of AQP3 expression can reduce H_2_O_2_ inflows induced by growth factors and weaken the signal cascade in cancer cells 20. It is possible that AQP3 plays a role in facilitating the transport of NOX-derived H_2_O_2_ signals stimulated by growth factors 24, and this may, to some extent, influence the downstream intracellular effects of H_2_O_2_ across biological barriers through AQP3. Currently, the mechanism by which AQP3 regulates H_2_O_2_ transport to promote cervical cancer's malignant progression remains unknown and requires further investigation.

In this study, we utilized cervical cancer HeLa cell line and established a xenograft tumor model in nude mice, and then we investigated the role and mechanism of AQP3 in the invasion and metastasis of cervical cancer by controlling NOX4-derived H_2_O_2_ signaling. The results indicate that NOX4-derived H_2_O_2_ transmembrane transport induced by growth factors is regulated by AQP3, and AQP3-dependent H_2_O_2_ signaling activates intracellular Syk/PI3K/Akt signaling cascades that promote the invasion and metastasis of cervical cancer.

## 2. Materials and Methods

### 2.1 Cell lines

Human cervical cancer cell lines HeLa (RRID: CVCL_0030), SiHa (RRID: CVCL_0032), C-33A (RRID: CVCL_1094), and human cervical epithelial immortalized cell line H8 (RRID: CVCL_9389) were obtained from the Pathology Laboratory of Xinjiang Medical University. All experiments were performed with mycoplasma-free cells and all cells have been authenticated using STR profiling. All cells were cultured in Dulbecco's modified Eagle's medium (DMEM, HyClone, USA), which was supplemented with 10% heat-inactivated fetal bovine serum (FBS, Gibco, USA), 100 U/mL penicillin, and 100 μg/mL streptomycin (Gibco, USA) at 37 °C in 5% CO_2_.

### 2.2 Western blotting

Cells were lysed with radioimmunoprecipitation assay (RIPA, Solarbio, China) buffer containing protease inhibitors and phosphatase inhibitors on ice. Equal volumes of protein extracts were boiled for 5 min and separated by SDS-PAGE and then transferred onto polyvinylidene fluoride (PVDF, Sigma-Aldrich LLC.; 3010040001) mem-branes. The membranes were incubated at 4 °C with primary antibodies overnight with the following antibodies: AQP3 (#AF5222, Affinity, China), Phospho-Syk (Tyr525/526) (#2710s, Cell Signaling Technology, USA), Syk (#sc1240, Santa Cruz, USA), Phospho-PI3Kinase p85α (Y607) (#ab182651, Abcam, UK), PI3Kinase p85α (#AF6241, Affinity, China), Phospho-Akt (Ser473) (#9271s, Cell Signaling Technology, USA), Akt (#4691s, Cell Signaling Technology, USA), and mouse anti-β-Actin (#66009-1-Ig, Proteintech, China). On the next day, the membranes were washed five times with TBST, and incubated with HRP-conjugated secondary antibody for 1 h. Next, the membrane was rinsed 5 times with TBST and then visualized by the Western Bright ECL detection system (Bio-Rad, USA).

### 2.3 Cell Migration (Scratch Wound) Assay

The migration of cells after the TGF-β1 (#100-21, Peprotech, USA) treatment was test-ed by wound scratch assay. HeLa cells were cultured as confluent monolayers in 6-well plates, synchronized in 1% FBS for 24 h, and wounded by removing a 300~500 μm-wide strip of cells across the well with a standard 10 μL pipette tip, floating cells were removed by washing with PBS. Media containing 10% FBS without or with indicated concentrations of TGF-β1, were added to the wells and incubated for an addtional 0 h, 24 h. Five representative images of the scratched areas were photographed under microscope at 0 h, 24 h, respectively. Finally, Image J software (NIH, Bethesda, MD, USA) was used to calculate the wound area.

### 2.4 Cell invasion assay

Cell invasion assay was conducted in transwell chambers (Corning Incorporated, Corning, NY, USA). The chambers were pre-coated with 60 μL Matrigel (BD Biosciences, CA, USA) for cell invasion assay. Cells treated with different transfections were starved overnight and then seeded in the upper chamber with 1 × 10^5^ cells in 100 μL of FBS-free medium. Meanwhile, 600 μL of medium containing 10% FBS was added to the lower cavity. The cells were incubated with TGF-β1 or IGF-1 (#G49-A118, MedChemExpress, USA) for 24h, cells in the upper chamber were removed, and invading cells in the lower chamber were fixed with methanol for 30 min, stained with crystal violet solution, and counted under the microscope.

### 2.5 Colony formation assay

Cells were seeded in 6-well plates at 5× 10^2^ cells per well. After 24 h of incubation, the cells were incubated with TGF-β1 for 2 weeks until a clone was visible with the naked eye. After that, the cells were fixed and stained with methanol and crystal violet for 30 min and photographed. Clone formation rates were calculated using Image J software. Each experiment was performed in triplicate.

### 2.6 Intracellular H_2_O_2_ detection

A DCFH-DA-tagged fluorescence (DCF) probe (#E004-1-1, Njjcbio, China) was used to test intracellular H_2_O_2_. A fluorescence microplate reader was used to test DCF intensity. Select HeLa cervical cancer cells, 2. 5 g /L trypsin Digestion. Termination of digestion. Cells were washed with serum-free medium and finally treated with containing Cells were resuspended in 10% neonatal fetal bovine serum medium. Adjust the whole cell number was 3 × 10^5^ cells /mL, seeded in 6-well plates, and cultured for 24 h. After that, a new medium was replaced. The cells in the normal and control groups and the treatment groups were treated with DPI (1μM, #43088, Sigma-Aldrich, Germany) or NAC (5mM, #A7250, Sigma-Aldrich, Germany), and then the cells were treated with 5 ng /mL TGF-β1 for 6 h. DCF (10 μM) was added to cells in a culture dish, which were incubated for 20 minutes at 37°C and then washed three times with serum-free cell culture medium to remove extracellular DCF. A fluorescence microplate reader was used to determine fluorescence with an excitation wavelength of 488 nm and an emission wavelength of 525 nm.

### 2.7 Cell transfection

For shAQP3 transfection, the Lentiviral vector containing the GFP and puromycin sequences was used to construct AQP3 shRNAs. Lentivirus was used to envelop AQP3 shRNA and transfected into HeLa cells for 16 hours. Subsequently, expression of the GFP gene was observed under a fluorescence microscope and screened with puromycin. The expression levels of target genes were examined by real-time PCR or western blotting as described below.

### 2.8 Gene expression by qRT-PCR

Total RNA was extracted from HeLa cells using the RNeasy Plus Mini Kit (TransGen, China). Multiscribe Reverse Transcriptase and random primers (Applied Biosystems) were used to synthesize cDNA from 1 ug of RNA. Samples were analyzed in duplicate by qRT-PCR (7500 Fast Real-Time PCR System, Bio-Rad, USA) using Sybr green chemistry and pairs of forward and reverse primers. The expression of genes of interest was normalized to the expression of GAPDH which was not affected by genotype. Data were analyzed using the 2^-ΔΔCT^ method for quantification.

### 2.9 Xenografts and tumor growth analysis *in vivo*

BALB/C nude mice (female, 3-5 weeks old, Slaccas, Shanghai, China) were bred under aseptic conditions and housed under a constant humidity of 60%-70% and room temperature of 18-20°C. The mice were used by the protocols approved by the Animal Care and Use Committee of Xinjiang Medical University. 4x10^6^ per nude mice subcutaneously, 1x10^6^ per nude mice in tail vein. The mice were then randomly assigned into the following four groups: vector, shAQP3, DPI, and NAC, and were injected subcutaneously or via the tail vein with shRNA-transfected HeLa cells. DPI (10 ng/kg), NAC (100 mg/kg), or PBS was administered by intraperitoneal injection (i.p.) once daily for 15 days according to the schedule. Mice were monitored daily to determine their body weight. To calculate the tumor volume, the following formula was used: tumor volume = [L × W^2^]/2, where W = tumor width and L = tumor length.

### 2.10 Histopathology and immunostaining

For histological evaluation, the dissected cervical tumors were fixed in 10% neutral-buffered formalin for approximately 1 week, embedded in paraffin, and sectioned. Samples were stained with hematoxylin and eosin (H&E).

For the immunohistochemical (IHC) staining, the tissue slides were deparaffinized with xylene (Zsbio, China) and rehydrated with ethanol. After inhibiting the endogenous peroxidase using 3% H_2_O_2_ in methanol, the sections were rinsed with PBS and the slides were blocked with 10% Normal Goat Serum (Zsbio, China) for 30 min at 20-25 °C, and were then incubated with primary antibodies (p-Syk, 1:100, p-p85α, 1:100, p-Akt, 1:100) overnight at 4 °C and then the secondary antibodies at room temperature for 30 min. Following DAB coloration, hematoxylin counterstaining, dehydration, and clearing in xylene, the slides were mounted. Using Image Pro Plus 6.0, staining degree was assessed by calculating the staining intensity and the positive area fraction. The staining intensity was assessed by calculating the cumulative optical density value (integrated option density, IOD). Staining degree was represented by the mean density value (mean density), which was Average Optical Density (AOD). AOD =Integral Optical Density (IOD)/ Positive area fraction.

### 2.11 Co-immunoprecipitation (Co-IP)

After the concentration of proteins was adjusted to equal incorporation, the lysate was immunoprecipitated with respective antibodies (GFP-tag, #T0005, Affinity, China, NOX4, #BM4135, Boster Bio, USA) or IgG for 2 h and then incubated with protein A/G agarose beads (Thermo Fisher, USA) at 4 °C overnight. Next, immunoprecipitated proteins were washed with Lysis buffer and eluted from agarose beads with 4×loading buffer. Bound proteins were then denatured and separated by western blot analysis.

### 2.12 Statistical analysis

Data are presented as mean ± standard deviation (SD). *P* values < 0.05 were considered to be statistically significant. Differences were analyzed using Student's t-test or one-way ANOVA. All statistical analyses were performed using SPSS 26.0 software.

## 3. Results

### 3.1. TGF-β1-induced production of exogenous H_2_O_2_ is transported into the cell via AQP3

**Figure 1:**
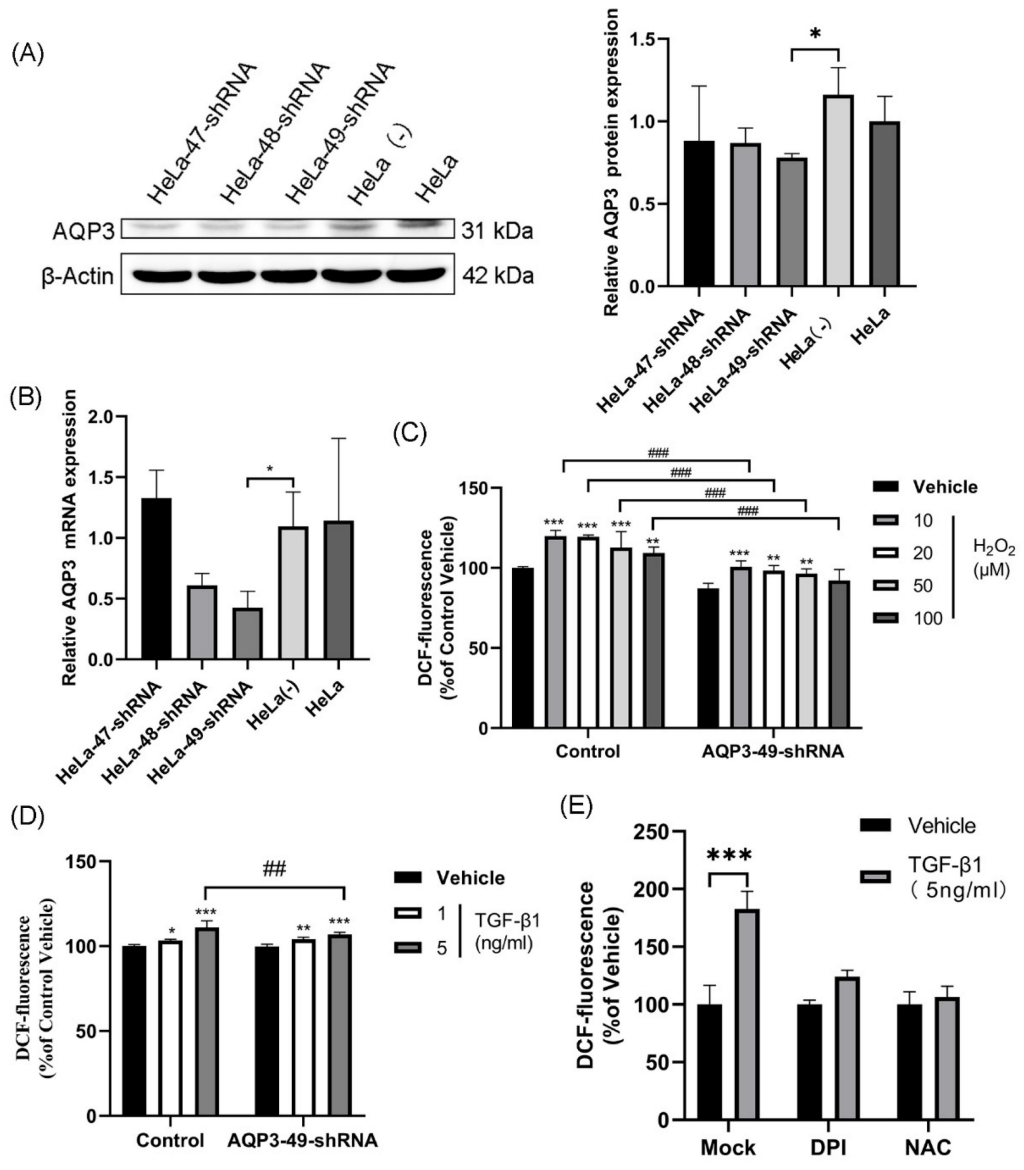
** TGF-β1-induced production of exogenous H_2_O_2_ is transported into the cell via AQP3.** (A) Western blotting was used to detect AQP3 protein expression in HeLa cells infected with lentiviral control (sh-control)- or AQP3-shRNA (sh-AQP3). β-Actin was used as a loading control. (B) AQP3 mRNA levels in HeLa cells infected with lentiviral control (sh-control)- or AQP3-shRNA (sh-AQP3) were detected by real-time fluorescent quantitative PCR. GAPDH was used as a loading control. (C) Control or AQP3-49-shRNA cells were incubated with different concentrations of H_2_O_2_ for 30 minutes, and the fluorescence value of intracellular DCF labeling, representing the intracellular H_2_O_2_ content, was detected by a fluorescent microplate reader. Compared to vehicle, *, control versus AQP3-49-shRNA, #. (D) The cells were incubated with different concentrations of TGF-β1 for 4 hours, and the intracellular H_2_O_2_ content was detected by a fluorescence microplate reader. Compared to vehicle, *, control versus AQP3-49-shRNA, #. (E) Cells were treated with DPI (10 μM, 6 h) or NAC (5 mM, 6 h), and the effect of TGF-β1 (5 ng/ml, 4 h) on intracellular H_2_O_2_ content was determined using a fluorescent microplate reader. Data were presented as mean ± SD. *, *P* < 0.05, **, *P* < 0.01, ***, *P*<0.001, ##, *P* < 0.01, ###, *P* < 0.001.

In our study, we selected HeLa cells as the target due to the high expression of AQP3 (Supplementary Table S1, Figure S1). To investigate AQP3's role in cervical cancer, we employed lentiviral shRNA to knock down AQP3 in HeLa cells. This resulted in a reduction in histone expression and mRNA levels compared to the control group (Figure 1A, B), with AQP3-49-shRNA showing the highest knockdown efficiency, leading us to select it for further experiments. To confirm the translocation of extracellular H_2_O_2_ into the cell via AQP3, we applied varying concentrations of H_2_O_2_ outside the cell, labeled it with the H_2_O_2_ active fluorescent dye DCFH-DA, and measured the fluorescence signal using a fluorescence labeling instrument to indicate intracellular H_2_O_2_ content. The results (Figure 1C) demonstrate a significant increase in intracellular H_2_O_2_ levels after H_2_O_2_ addition, with the control group showing notably higher levels compared to the AQP3-49-shRNA group (Figure 1C). Likewise, the introduction of TGF-β1, a NOX4 stimulator, markedly enhanced intracellular H_2_O_2_ transport, and AQP3 knockdown inhibited the extracellular H_2_O_2_ transport compared with the control group (Figure 1D). These results indicate that AQP3 can transport extracellular H_2_O_2_ into cells. Next, NOX4 inhibitor diphenyleneiodonium (DPI) and H_2_O_2_ inhibitor N-Acetyl-L-cysteine (NAC) were incubated with HeLa cells, and after TGF-β1 stimulation, we found that Pretreatment with DPI and NAC significantly reduced the intracellular H_2_O_2_ level induced (Figure 1E). We therefore demonstrate that TGF-β1 stimulation induces exogenous, NOX4-produced H_2_O_2_ translocation into the cell via AQP3.

### 3.2. Knockdown of AQP3 attenuated the effects of NOX4-derived H_2_O_2_ on migration, invasion, and proliferation of HeLa cells

**Figure 2:**
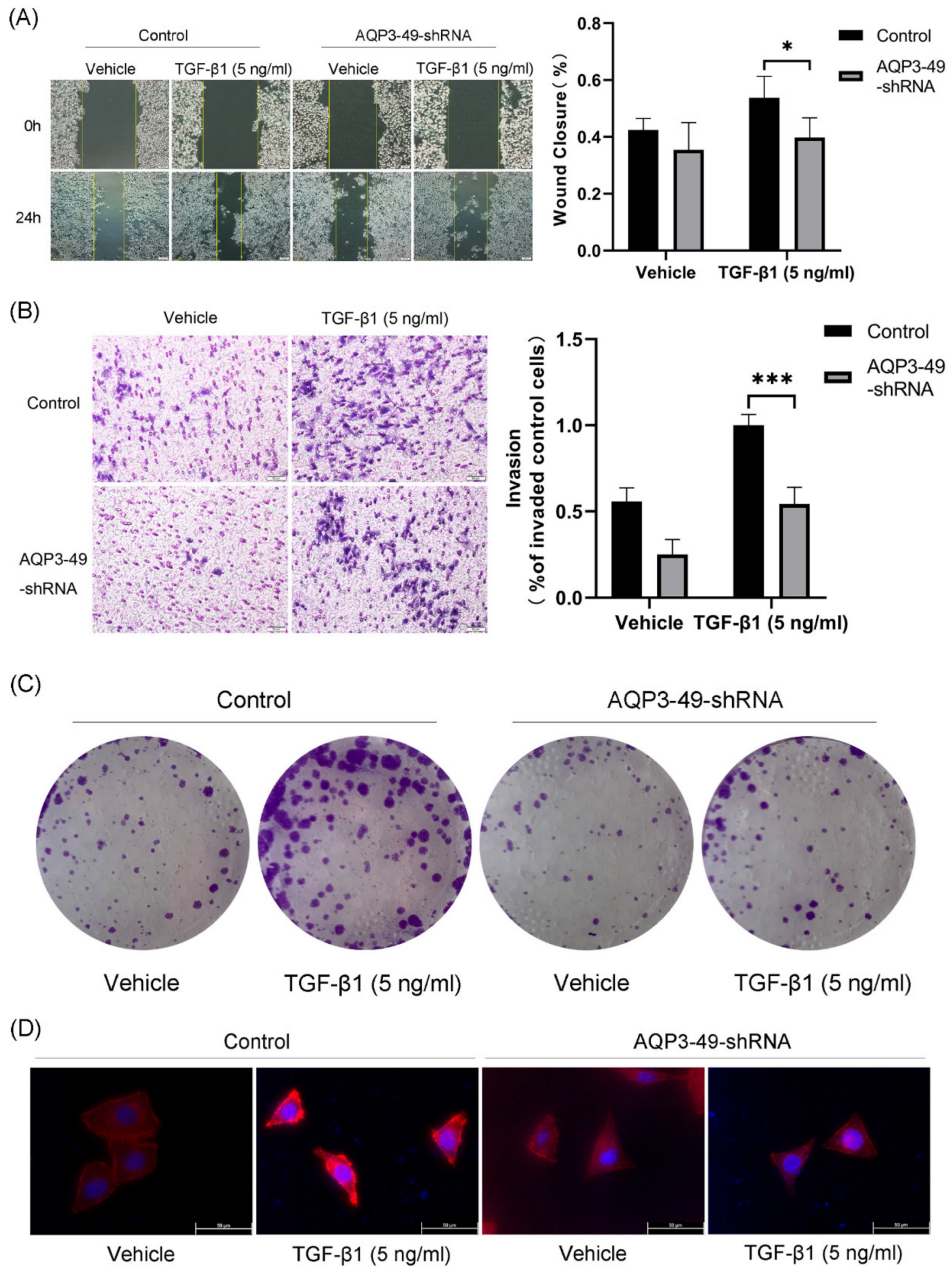
** Knockdown of AQP3 attenuated the effects of NOX4-derived H_2_O_2_ on migration, invasion, and proliferation of HeLa cells.** (A) Scratch was made in 6-well plates, and 24 h later, fluorescence microscopy was used to examine the migration of control or AQP3-49-shRNA cells in response to TGF-β1 training. (B) The invasion efficiency of control and AQP3-49-shRNA HeLa cells under TGF-β1 was examined with an 8-μm-pore-size transwell chamber. (C) Colony formation assays were used to test the effects of TGF-β1 on HeLa cell growth. (D) Fluorescence microscopy analysis of visualized F-actin polymerization of control or AQP3-49-shRNA cells with TRITC Phalloidin after TGF-β1 stimulation (5 ng/ml, 30 min). Data were presented as mean ± SD. *, *P* < 0.05, ***, *P* < 0.001.

Multiple studies have shown that AQP3 promotes cancer progression by increasing the motility and invasiveness of cancer cells 25. Therefore, we first investigated the ability of AQP3 to promote cell migration by transporting H_2_O_2_. Cell scratch assay was used to test the wound closure rate in HeLa cells. The results showed that the knockdown of AQP3 significantly inhibited the wound healing ability (Figure 2A). We next studied the impact of AQP3 on the invasion capability of cervical cancer cells. The results showed that the knockdown of AQP3 in HeLa cells resulted in a suppressive effect on cell invasion, even when exogenous NOX4-derived H_2_O_2_ was produced by the addition of TGF-β1 (Figure 2B). In parallel, the colony formation assay further sup-ported the oncogenic role of AQP3 (Figure 2C).

In the tumor microenvironment, various signals, including hypoxia, promote migration by enhancing cytoskeletal activity 26. F-actin is one of the most important structural components of the cytoskeleton, whose assembly is closely related to cell migration. Accordingly, we next used phalloidin to label F-actin to observe changes in the cytoskeleton upon migration. In the presence of TGF-β1, the polarized morphology of F-actin during migration was observed at the cell edge in control cells, but not in AQP3 knockdown cells (Figure 2D). In conclusion, these findings strongly link AQP3 to the metastatic and invasive traits of cervical cancer cells.

### 3.3. AQP3 regulates Syk phosphorylation and activation of PI3K/Akt signaling path-way in HeLa cells

**Figure 3:**
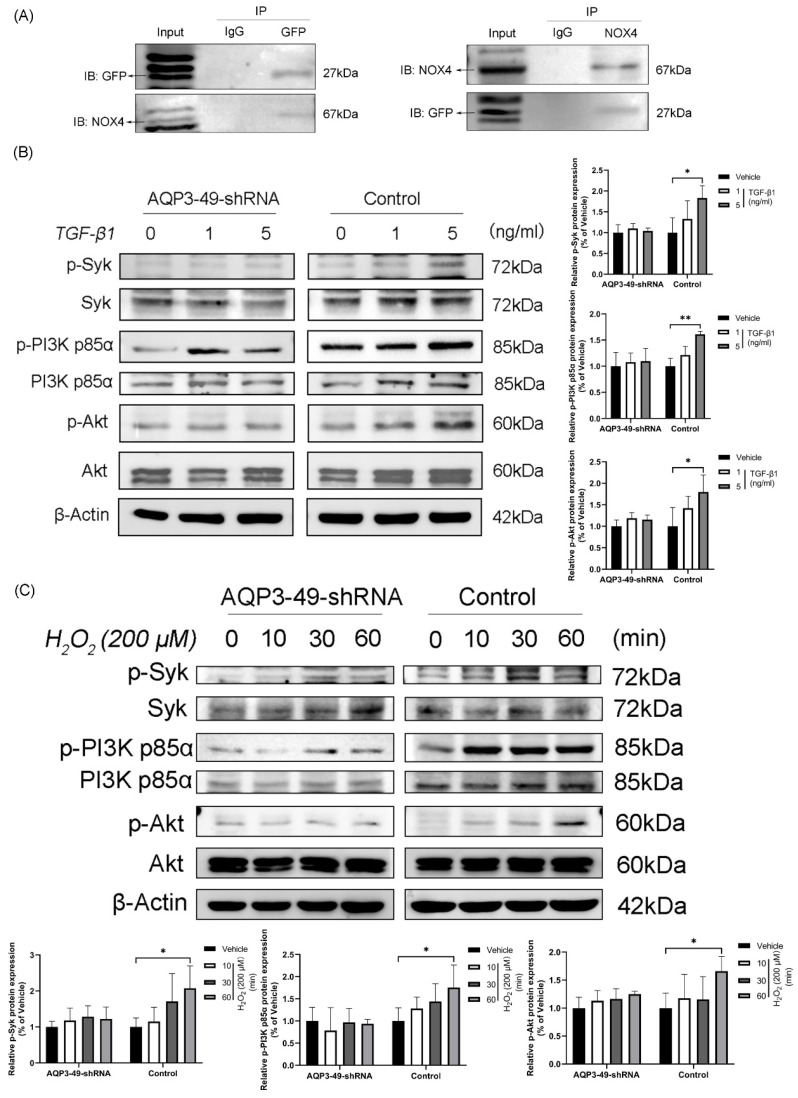
** AQP3 regulates Syk phosphorylation and activation of the PI3K/Akt signaling pathway in HeLa cells.** (A) Collection of lentiviral control (sh-control) HeLa cells loaded with Green Fluorescent Protein Tag (GFP), CO-Immunoprecipitation detection analyzes the interaction of AQP3 and NOX4 complexes in HeLa cells. (B) Control and AQP3-49-shRNA HeLa cells were treated with different concentrations of TGF-β1 for 30 minutes, and the phosphorylation levels of Syk and PI3K/Akt were analyzed by western blot. (C) Western blot was used to detect the expression of Syk, PI3K p85α, and Akt phosphorylation in control and AQP3-49-shRNA HeLa cells treated with 200 μM H_2_O_2_ at different times. β-Actin was used as a loading control. Data were presented as mean ± SD. *, *P* < 0.05, **, *P* < 0.01.

To uncover the molecular mechanism underlying the oncogenic effects of AQP3 in cervical cancer cells, we found by immunoprecipitation experiments that AQP3 inter-acts with NOX4 and may co-form complexes at the cell membrane (Figure 3A).

Next, we examined whether changes in AQP3 expression affected the PI3K/Akt-related signaling pathways, given previous suggestions of AQP3's role in cancer progression through this pathway 27, 28. As shown in Figure 3B, the knockdown of AQP3 in HeLa cells with different concentrations of TGF-β1 reduced the levels of phosphorylated PI3K p85α and Akt, while the total protein expression level was almost unchanged. Given that Spleen Tyrosine Kinase (Syk) is known to phosphorylate the PI3K signaling pathway by initiating BCR signaling 29, we investigated Syk's expression. Western blot results (Figure 3B) revealed increased phosphorylation of Syk with rising TGF-β1 concentrations, and this effect was significantly suppressed by AQP3 knockdown. Subsequently, we observed that the addition of exogenous H_2_O_2_ could activate PI3K signaling pathway in a time-dependent manner and that AQP3 knockdown cells similarly attenuated the activation of phosphorylated proteins (Figure 3C). These findings suggest that AQP3 may exert its tumorigenic function in cervical cancer cells by activating PI3K/Akt-related signaling pathway.

### 3.4. Increased H_2_O_2_ after TGF-β1 stimulation promotes phosphorylation of Syk, PI3K p85α and Akt

**Figure 4:**
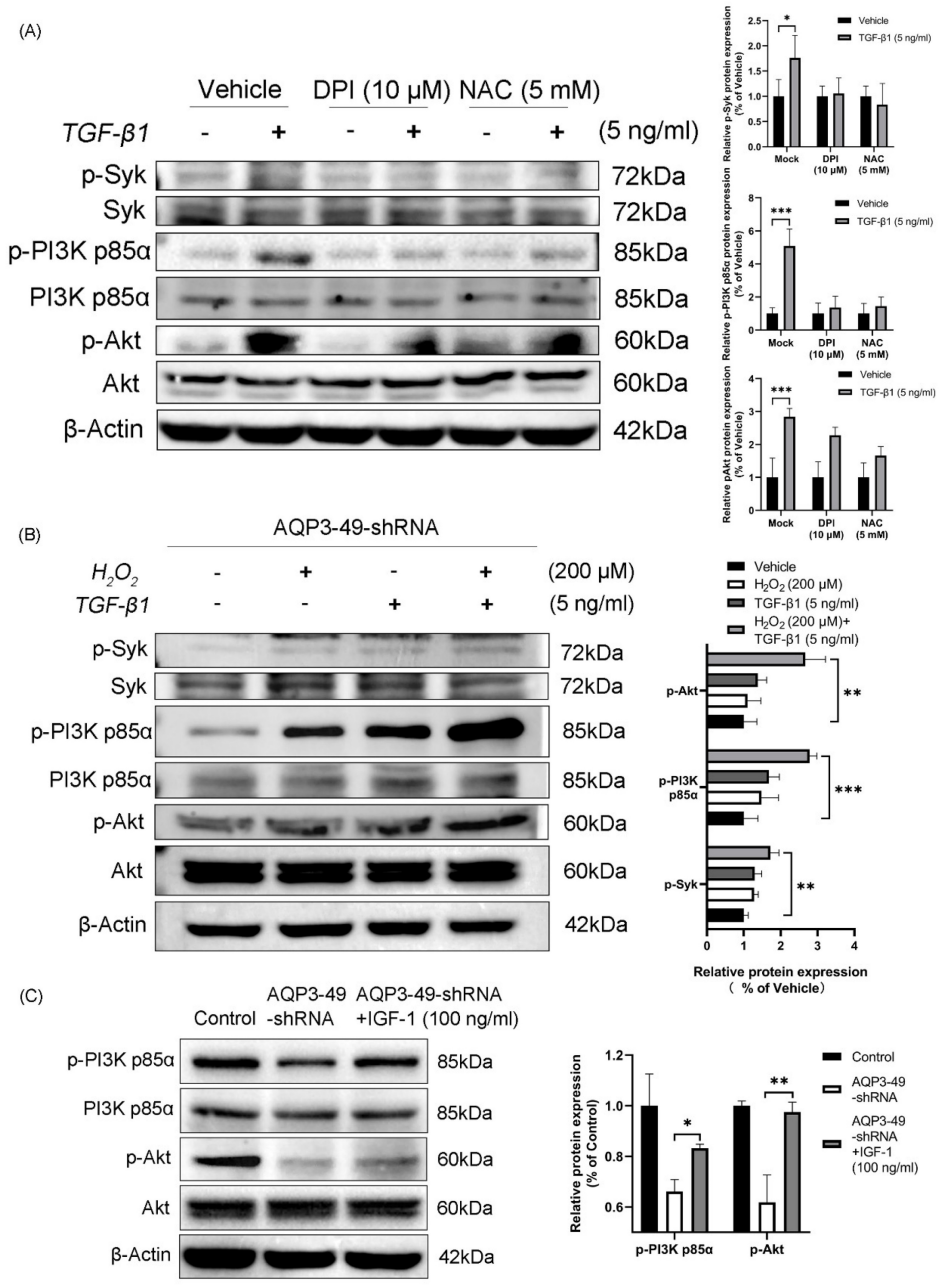
** H_2_O_2_ generated by TGF-β1 stimulation promotes phosphorylation of Syk, PI3K p85α, and Akt.** (A) HeLa cells were treated with DPI (10 μM, 6 h) or NAC (5 mM, 6 h), and the phosphorylation of Syk, PI3K p85α and Akt in response to TGF-β1 (5 ng/ml, 30 min) was detected by western blot. (B) AQP3-49-shRNA HeLa cells were treated with 200 μM H_2_O_2_ and TGF-β1 (5 ng/ml) alone or in combination for 30 min. Western blot analysis was then performed. (C) Western blot was used to analyze the expression changes of PI3K/Akt signaling pathway proteins in AQP3-49-shRNA HeLa cells treated with IGF-1 (100 ng/ml). β-Actin was used as a loading control. Data were presented as mean ± SD. *, *P* < 0.05, **, *P* < 0.01, ***, *P* < 0.001.

To investigate whether NOX4-derived H**_2_**O**_2_** promotes PI3K/Akt phosphorylation, HeLa cells were pretreated. Cells incubated with DPI and NAC showed a diminished response to TGF-β1 and decreased phosphorylation compared with controls (Figure 4A). This suggests that H**_2_**O**_2_** produced by NOX4 is involved in the expression of PI3K-related signaling pathway under TGF-β1 stimulation. AQP3 knockdown cells were treated with TGF-β1 alone, H**_2_**O**_2_** alone or in combination with TGF-β1. The results demonstrated that H_2_O_2_ alone induced Syk, PI3K p85α, and Akt phosphorylation in the tested cell line. Furthermore, adding H_2_O_2_ to TGF-β1 increased the H_2_O_2_-induced phosphorylation of Syk, PI3K p85α, and Akt (Figure 4B). We next investigated whether insulin like growth factor-1 (IGF-1), an agonist of PI3K, could reverse the effects of AQP3 knockdown on the phosphorylation of PI3K/Akt pathway. After 100 ng/ml IGF-1 stimulation was given to AQP3 knocked down cells, and Western blot was performed. The results showed that IGF-1 significantly increased PI3K p85α and Akt phosphorylation in AQP3-49-shRNA HeLa cells, while the total protein was relatively unchanged (Figure 4C). It is suggested that knockdown of AQP3 inhibits the activation of PI3K/Akt signaling pathway in cervical cancer HeLa cells, and this effect can be partially reversed by PI3K agonists. We then looked at whether IGF-1 could induce migratory and invasive behavior. As supported, our data showed that although AQP3 knockdown reduced H_2_O_2_ signaling pathway conduction, IGF-1 enhanced cell wound healing abilities and invasion abilities (Supplementary Figure S2A, B). Similarly, a polarized morphology of F-actin during migration was observed at the edge of IGF-1-treated cells (Supplementary Figure S2C). These results confirm that AQP3 regulates downstream signaling through NOX4-derived H_2_O_2_ induced by TGF-β1 stimulation. Additionally, the PI3K agonist (IGF-1) partially reversed the inhibition of the PI3K/Akt pathway caused by AQP3 knockdown, providing further evidence of AQP3's regulatory role in NOX4-derived H_2_O_2_ signaling. This, in turn, leads to the activation of the PI3K/Akt pathway, contributing to the malignant progression of cervical cancer.

### 3.5. Knockdown of AQP3 inhibited the formation of subcutaneous xenograft tumors in nude mice

**Figure 5:**
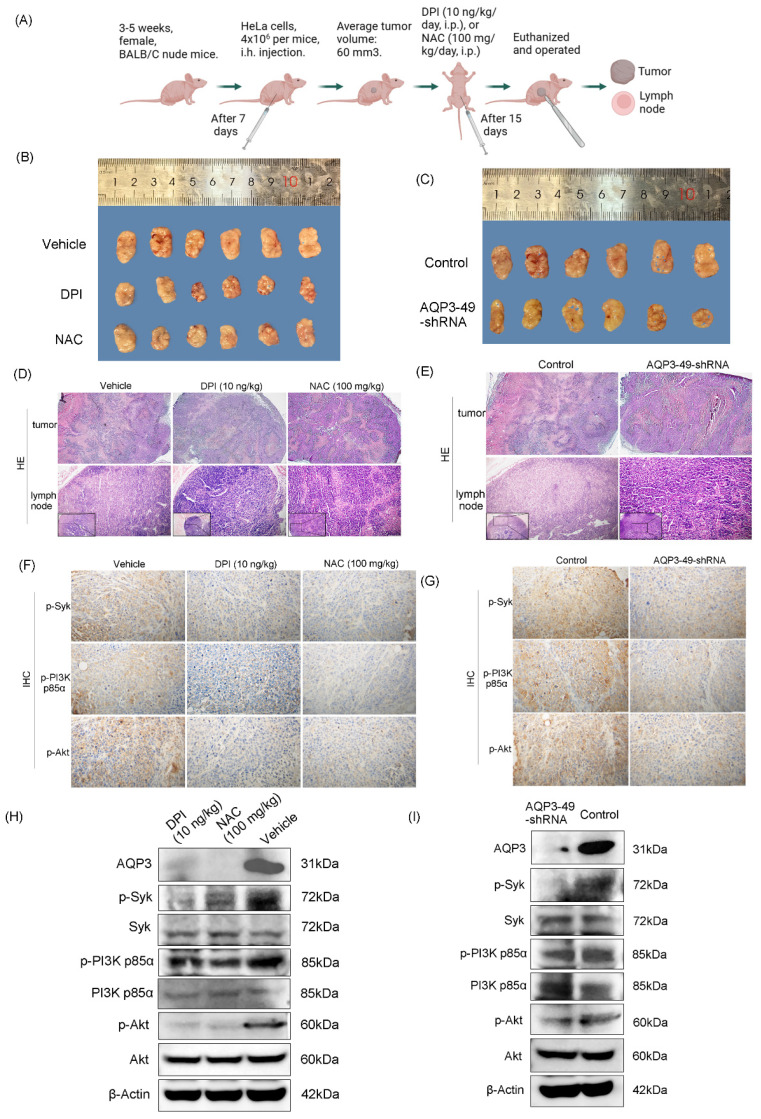
** Knockdown of AQP3 inhibited the formation of subcutaneous xenograft tumors in nude mice.** (A) Pattern diagram of subcutaneous xenograft tumor nude mice. (B) Effect of DPI (10 ng/kg) and NAC (100 mg/kg) on subcutaneous tumor growth volume. (C) The difference in transplanted tumor volume between the control group and the AQP3-49-shRNA group. (D) (E) H.E. staining analysis of tumor tissue (40 x) manifestation and lymph node (100 x and 200 x) metastasis rate in different groups. (F) After DPI and NAC treatment, the expression of p-Syk, p-PI3K p85α, and p-Akt were detected by immunohistochemistry (IHC) (400 x). (G) IHC (400 x) analysis of the effect of AQP3-49-shRNA on phosphorylation expression in mice. Average Optical Density (AOD) = Integral Optical Density (IOD)/ Positive area. (H) Western blot analysis of the effects of DPI and NAC on the expression of key proteins in nude mice. (I) Western blot was used to detect the expression changes of key proteins in the AQP3-49-shRNA group. n = 6/group.

Our previous research found that AQP3 expression in carcinoma of the cervix significantly increased in advanced stage disease, and patients with deeper tumor infiltration, lymph node metastases or larger tumor volume, which suggests AQP3 may participate in the initiation and progression of cervical carcinoma by promoting tumor growth, invasion or lymph node metastasis 30. As mentioned earlier, the knockdown of AQP3 showed a protective effect against cervical cancer progression. Next, AQP3 knockdown HeLa cells or control cells were injected into nude mice to establish a subcutaneous xenograft model (Figure 5A). The control model was treated with PBS, DPI (10 ng/kg, i.p, 15 days) or NAC (100 mg/kg, i.p, 15 days). It was found that DPI or NAC treatment slowed tumor growth compared to vehicle (Figure 5B), and there was no significant reduction in the body weight (Supplementary Figure S3A). At the same time, compared with the control group, the knockdown of AQP3 also slowed the growth of subcutaneous tumors in nude mice (Figure 5C), while no significant difference in body weight was observed (Supplementary Figure S3B). After observing the Hematoxylin and Eosin staining (H.E.) sections of the tumors, it was found that the necrotic area in both PBS (vehicle) group and the control was larger than that of both DPI or NAC-treated group and the AQP3 knockdown. It indicates that they all have relatively high degrees of malignancy (Figure 5D, E, top). Since advanced cervical cancer is prone to lymph node metastasis, we also examined lymph node metastasis in nude mice.

In our results, the rate of lymph node metastasis was reduced in DPI- or NAC-treated groups, as well as in the AQP3 knockdown nude mice (Figure 5D, E, bottom, Supplementary Figure S3C). Then Immunohistochemistry (IHC) of xenograft tumors was conducted to assess p-Syk, p-PI3K p85α, and p-Akt *in vivo*. The results showed that treatment with DPI or NAC, as well as knockdown of AQP3, significantly inhibited the protein expression of p-Syk, p-PI3K p85α or p-Akt (Figure 5F, G, Supplementary Figure S3D). We also evaluated the effect of DPI or NAC on the above protein expression by Western blot and found that AQP3, p-Syk, p-PI3K p85 or p-Akt levels were down-regulated (Figure 5H, Supplementary Figure S3E). Western blot results also showed that AQP3 knockdown decreased the phosphorylation of the key proteins (Figure 5I, Supplementary Figure S3F).

### 3.6. AQP3 transport of H_2_O_2_ enhances *in vivo* metastasis in nude mice

**Figure 6:**
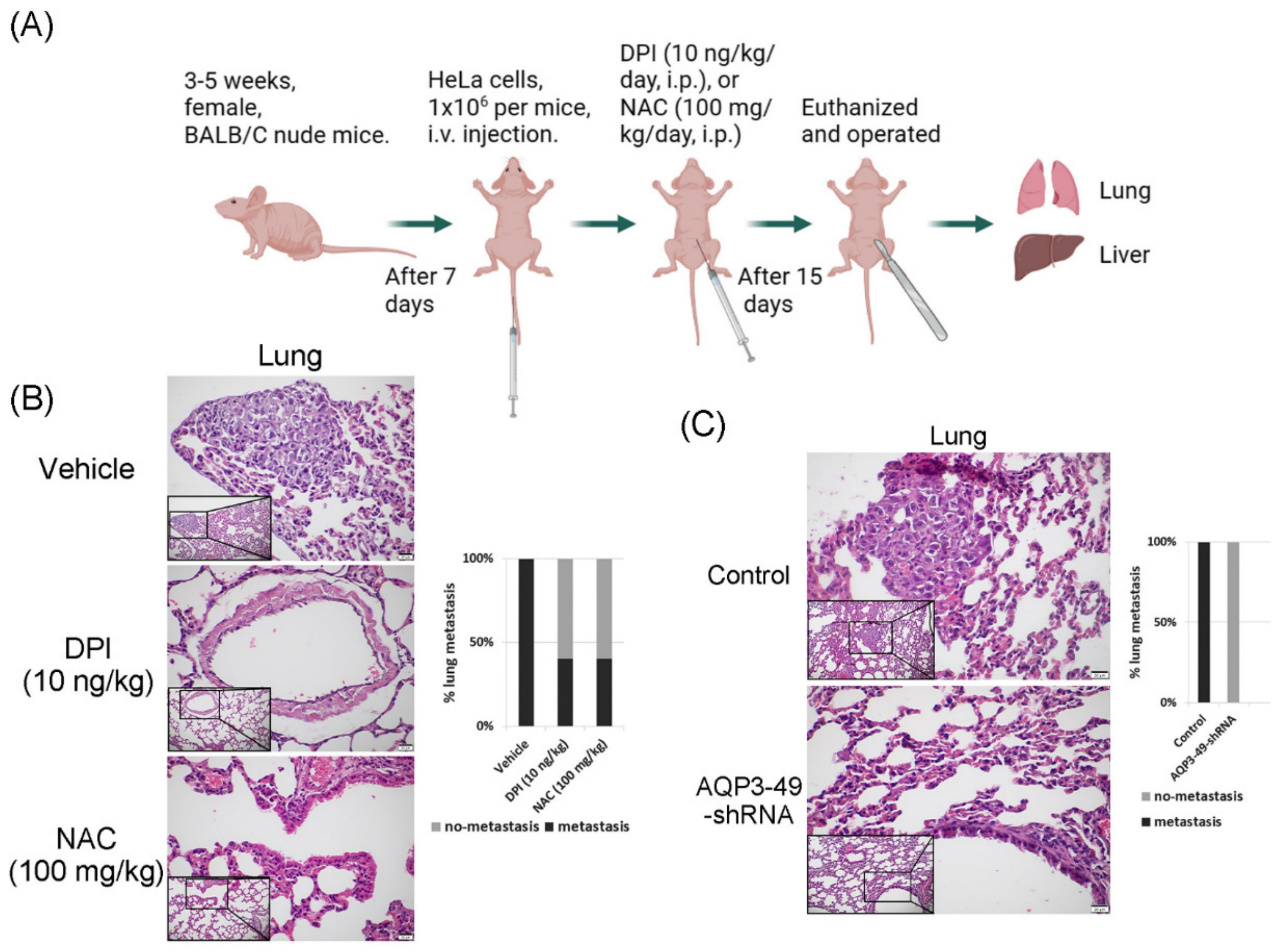
** AQP3 transport of H_2_O_2_ enhances *in vivo* metastasis in nude mice.** (A) Modal pattern of tail vein injected nude mice. (B) H.E. staining analysis of the effect of treatment with DPI and NAC on the rate of lung metastasis in nude mice *in vivo*. The small image below left is 200x, the enlarged image is 400x. (C) Detection of the impact of AQP3 on the metastasis of HeLa cells to the lungs by H.E. staining assay. The small image below left is 200x, the enlarged image is 400x. n = 5/group.

To explore the effect of AQP3 on the transcellular transport of NOX4-produced H_2_O_2_ in nude mice *in vivo*, we constructed a tail vein metastasis model (Figure 6A). For tail vein-injected nude mice, no significant difference in body weight could be observed prior to execution (Supplementary Figure S4A, B). We euthanized the mice 15 days after DPI or NAC application and then removed the lungs and liver to observe metastasis. H.E. staining results showed that DPI or NAC could significantly attenuate the lung metastasis from a tail vein injection of HeLa cells (Figure 6B). The knock-down of AQP3 also had the same effect, and the control nude mice had more lung metastatic foci, no pulmonary metastases were detected in AQP3 knockdown group. (Figure 6C). In addition, we found the presence of liver metastases in or near the hepatic blood sinusoids. However, no significant difference was found in the liver metastasis among the groups (Supplementary Figure S4C, D). Our data confirmed the role of AQP3 in promoting HeLa cell metastasis *in vivo*. In summary, the reduction of NOX4-derived H_2_O_2_ by DPI or NAC, or the attenuation of AQP3 as the ROS transport channel, led to a decrease in tumor cell metastasis to the lungs. AQP3 was demonstrated to have a significant oncogenic role in ROS regulation.

## 4. Discussion

Inflammatory factors are essential components of the tumor microenvironment. Inflammation can not only promote cell proliferation and metastasis through epigenetics and abnormal gene expression, angiogenesis, etc. but also release lots of reactive oxygen species (ROS) that promote cancer evolution 31. Hence, ROS play a crucial role rather than serving merely as by-products of REDOX reactions induced by oxidative stress. ROS are a class of highly reactive free radicals, such as hydroxyl radical (•OH), the superoxide radical (O2•-), and hydrogen peroxide (H_2_O_2_) 32, 33. Superoxide can rapidly and spontaneously convert to H_2_O_2_, which serves as a signaling molecule, leading to the abnormal activation of various signaling pathways and contributing to cancer progression.

An important clinicopathological feature of cervical cancer is often accompanied by persistent chronic inflammation. Considering the significant role of ROS in pathophysiology, regulating the entry of extracellular ROS produced by NOX4 into cells to promote pro-cancer signaling in cervical cancer has become a pressing research concern. Several studies have shown that certain AQPs, such as AQP3, AQP5, AQP8, AQP9, can transport various polar small molecules, including the regulation of extracellular H_2_O_2_ transport, making them known as peroxiporins 34. Among them, AQP3 is well known to transport H_2_O_2_ in different studies. In this preliminary study, we found that the expression of AQP3 in HeLa cell line was highest among the 4 cell lines representing the main molecular subtypes of cervical cancer. Knock-down of AQP3 in HeLa cell line attenuated the entry of NOX4-derived H_2_O_2_ into the cells induced by TGF-β1 and the migration and invasive capacity of HeLa cells, and which was confirmed by the nude mouse xenograft tumor model. An important problem is how AQP3 regulates NOX4-derived H_2_O_2_? As far as the literature we have reviewed are concerned, this issue is rarely reported. Here, co-localization of AQP3 and NOX4 was observed through CO-IP on HeLa cell membrane, which suggested that there was an interaction between AQP3 and NOX4 and that TGF-β1 triggered NOX4 to produce more H_2_O_2_, a second messenger, flowing into the cell upon opening of AQP3 channel. And we explored whether the onset of AQP3 transporting H_2_O_2_ was related to NOX4, and verified the effect of AQP3 on promoting the invasion and metastasis after NOX4 activation in HeLa cells. AQP3 has been studied to influence cancer progression by transporting H_2_O_2_ and regulating intracellular ROS levels. AQP3 promotes malignant transformation and stimulates the proliferation and metastasis of lung adenocarcinoma cells by prompting the uptake of H_2_O_2_ to further oxidize and in-activate PTEN and inhibit autophagy 35. Additionally, silencing of AQP3 reduces MMP expression in gastric cancer cells and attenuates invasion and metastasis of gastric cancer cells through a PI3K/Akt-dependent manner 27. In breast cancer AQP3 has also been shown to regulate oxidative responses and PI3K/Akt activation, affecting its progression 28, 36. These studies all support our view that H_2_O_2_ acts as a second messenger to promote the progression of cervical cancer through the mediation of AQP3.

H_2_O_2_ signal is similar to other signal transduction, characterized by a series of phosphorylation events that occur locally in the cell. Syk (spleen tyrosine kinase), a non-receptor tyrosine kinase that mediates signaling downstream of a variety of transmembrane receptors, has been detected to be highly associated with malignant tumors including, but not limited to, lymphoid malignancies, colon cancer, non-small cell lung cancer, breast cancer, and ovarian cancer 29, 37-39. In our study, Syk phosphorylation was significantly enhanced by the entry of extracellular H_2_O_2_. In vivo and in vitro assays, Syk phosphorylation was reduced after the treatment of NOX4 inhibitor DPI and H_2_O_2_ inhibitor NAC. Previous studies have shown that 15(S)-HETE induces ROS production in XO-dependent activation of NOX, which leads to the activation of non-receptor tyrosine kinases (NRTK) such as Syk and Pyk2 in monocytes 40. Subsequent experiments demonstrated that ROS production enhanced atherogenesis by Syk and Pyk2-mediated STAT1 activation and CD36 expression 41. Coincidentally, in a study of periodontitis, the authors demonstrated that Trem2 increases intracellular ROS levels and mediates osteoclast differentiation through a SYK-dependent signaling cascade 42. Thus, Syk may function as a downstream molecule in response to H_2_O_2_ signaling.

Syk, as a downstream effector shared by multiple oncogenic receptors, mediates downstream signal transduction of multiple transmembrane receptors 43. We next explored the downstream pathways of H_2_O_2_/Syk signaling in more depth. In our experiments, when Syk was phosphorylated due to increased H_2_O_2_ entering into the cell, the PI3K/Akt signaling pathway was activated, promoting invasion and metastasis in HeLa. This result was also confirmed *in vivo* animal experiments, where nude mice treated with more H_2_O_2_ being translocated into the cells exhibited worse malignancy manifestations, like enlarged volume of graft tumors or increased number of metastatic foci. In other studies, it has been found that there are several other pathways involved in the delivery of Syk signaling. Both the chemical inhibition and molecular depletion of Syk induced the pro-apoptotic HRK protein via a PI3K/Akt-dependent mechanism in BCR-dependent DLBCL cell lines and primary tumors with low baseline NF-κB activity 44. Another finding showed that phosphorylation of CD19-Akt was only observed in the presence of Syk-wild-type but not Syk^K402A^-kinase-dead form 29. It is, therefore, possible that the PI3K/Akt pathway is located in the downstream signaling to function in response to Syk. PI3K/Akt is also a classic oncogenic pathway 45, which was activated in the H_2_O_2_/Syk signaling pathway. We validated the pro-cancer role of the H_2_O_2_/Syk/PI3K signaling axis. Our data demonstrates that inflammatory mediator TGF-β1 stimulates cervical cancer cells to accelerate metabolism, prompting NOX4 in the cell membrane to produce large amounts of ROS, which are converted to relatively stable H_2_O_2_. AQP3 interacts with NOX4 in the cell membrane, and a large amount of H_2_O_2_ flows into the cell through the open AQP3 channel, which acts as a signaling molecule to activate the Syk/PI3K/Akt pathway, promoting the invasion and metastasis of cervical cancer.

Transcription of the Syk gene produces two selective splice products: the full-length Syk, termed Syk (L), and the shorter gene product, SykB, also known as Syk (S). Current studies have shown that Syk (L) and Syk (S) have different effects on the growth characteristics of cancer cells. The ability of Syk to act as a promoter or re-pressor of malignant cell growth appears to be highly dependent on the cell type and its stage of differentiation, the relative levels of the two Syk isoforms expressed, etc. 46. Therefore, the role of Syk on the proliferation and migration of cancer cells requires further study.

Taken together, our results suggest that AQP3 promotes cervical cancer invasion and metastasis by regulating NOX4-derived H_2_O_2_ transport into cancer cells, thereby activating the Syk/PI3K/Akt signaling pathway. Inhibition of the H_2_O_2_/Syk/PI3K/Akt signaling axis may be a potentially effective way to treat cervical cancer, and AQP3 could be a potentially effective target for the treatment of cervical cancer. In addition, human cervical cancer tissue samples were not included in our study for the time being, which warrants further subsequent studies.

## Supplementary Material

Supplementary figures and table.

## Abbreviations

Akt: Protein Kinase B
AQP3: Aquaporin 3
DPI: diphenyleneiodonium
EMT: Epithelial-mesenchymal transition
GFP: Green Fluorescent Protein Tag
H_2_O_2_: hydrogen peroxide
H.E.: Hematoxylin and Eosin staining
IGF-1: Insulin-like growth factor 1
IHC: Immunohistochemistry
NAC: N-Acetylcysteine
NOX4: NADPH oxidase 4
PI3K: Phosphoinositide 3-kinase
ROS: reactive oxygen species
Syk: Spleen Tyrosine Kinase
TGF-β1: transforming growth factor-β1
TME: tumor microenvironment
