# Supplementary material for: AQP3 Promotes the Invasion and Metastasis in Cervical Cancer by Regulating NOX4-derived H2O2 Activation of Syk/PI3K/Akt Signaling Axis
Source: J Cancer. 2024 Jan 1;15(4):1124–37. doi: 10.7150/jca.91360 (PMC10788729; doi:10.7150/jca.91360)
Supplement: Supplementary file 1 — Supplementary figures and table. [file jcav15p1124s1.pdf]

Table S1. Molecular classification of human cervical cancer and epithelial immortalized cell lines.

Cell line	Tumor type	HPV16	HPV18
H8	-	-	-
C-33A	SC	-	-
SiHa	SC	+	
HeLa	AC	-	+

AC, adenocarcinoma; SC, squamous carcinoma.

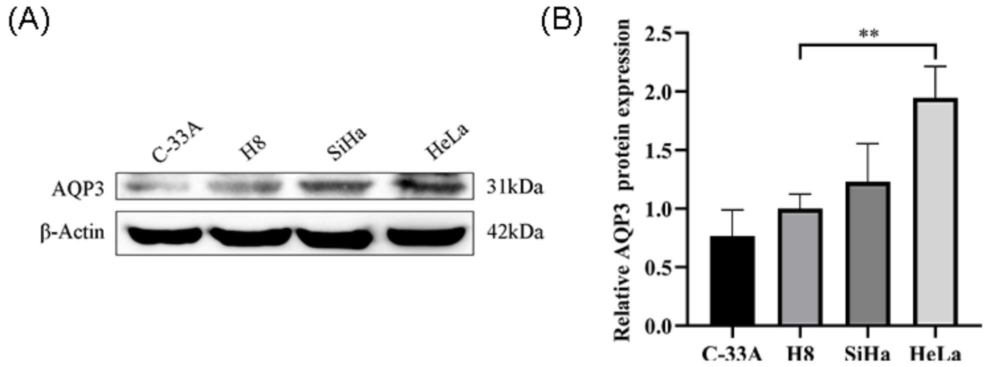

Figure S1. Relative expression of several cervical cancer cell lines and cervical immortalized cell line. (A) Western blot analysis of AQP3 expression in C-33A, H8, SiHa, and HeLa. (B) The gray value was quantified and plotted. β-Actin was used as a loading control. Data were presented as mean ± SD. **, $P < 0.01$.

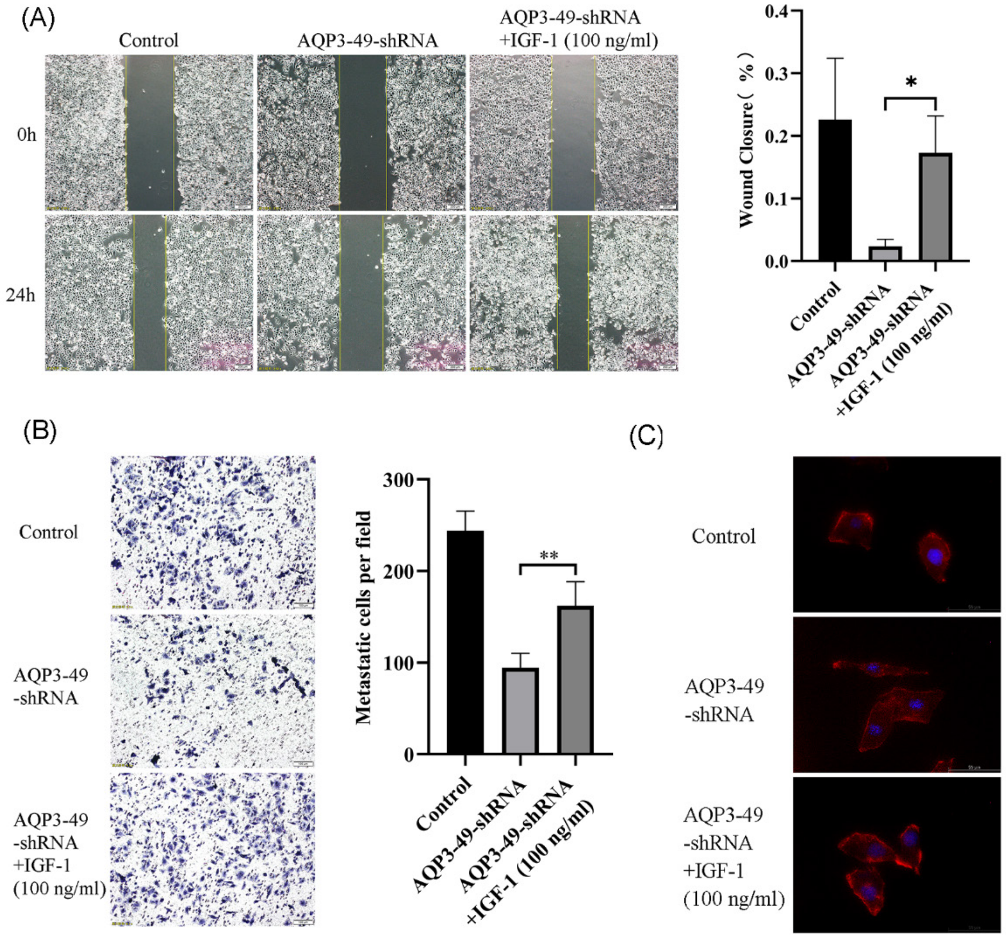

Figure S2. PI3K agonist (IGF-1) partially rescued the diminished invasive and migratory capacity caused by AQP3 knockout. (A) The difference in cell migration distance under the action of IGF-1 was detected by the scratch test. (B) Fluorescence microscopy of F-actin polymerization in cells by TRITC Phalloidin. (C) Differences in invasive ability following IGF-1 treatment. Data were presented as mean ± SD. *, $P < 0.05$, **, $P < 0.01$.

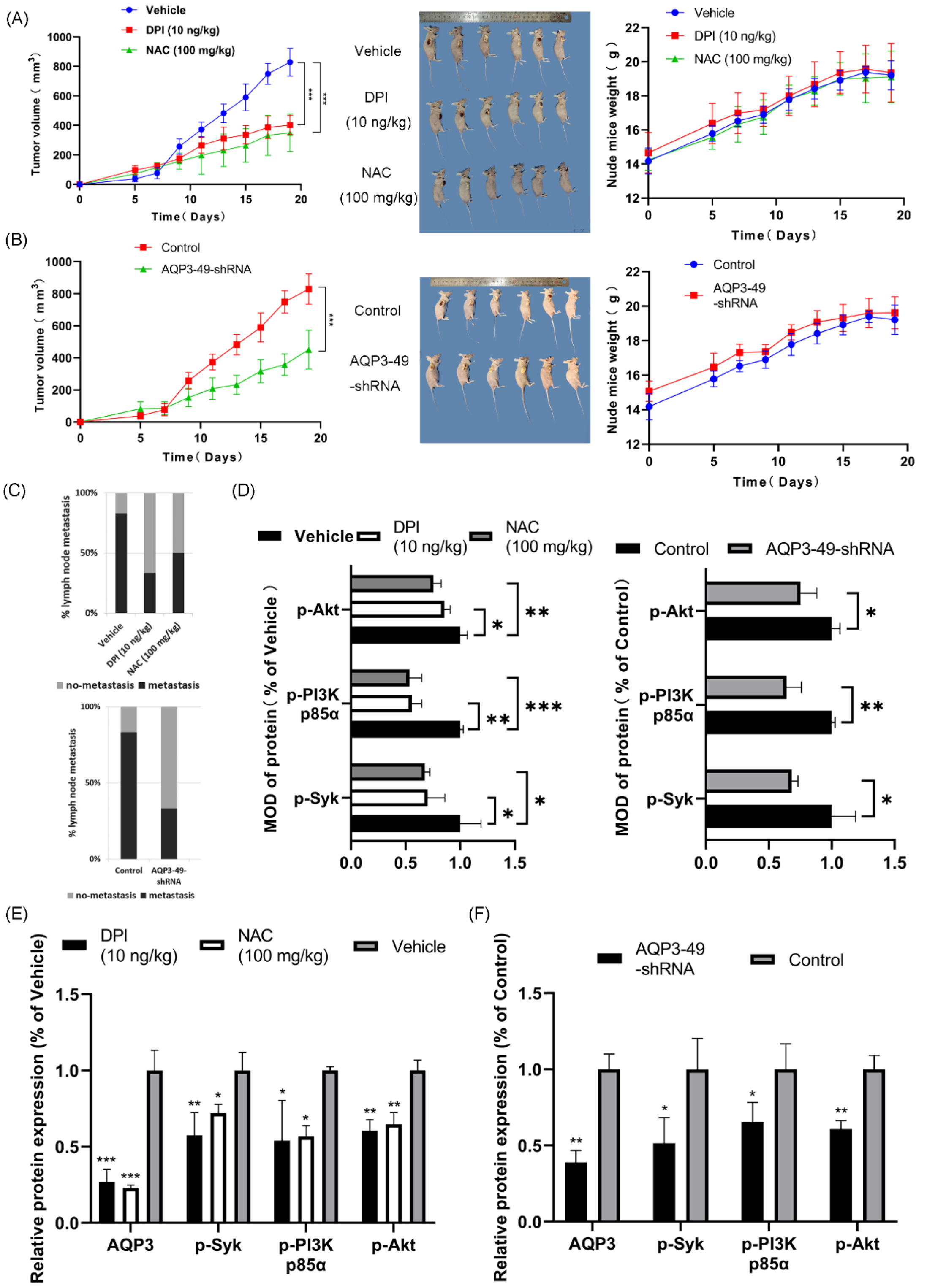

Figure S3. Body weight growth curve of nude mice with subcutaneous xenograft tumors. (A) Change curves of tumor volume and body weight growth in nude mice after DPI- and NAC-injection. (B) Tumor volume and body weight growth curves of AQP3 knockdown and control nude mice. (C) Lymph node metastasis rate. (D) Quantification of positivity for immunohistochemistry, expressed as Average Optical Density (AOD) values, Average Optical Density

(AOD) = Integral Optical Density (IOD)/ Positive area. (E) (F) Quantitative data for Western blot. Data were presented as mean ± SD. *, $P<0.05$, **, $P<0.01$, ***, $P<0.001$.

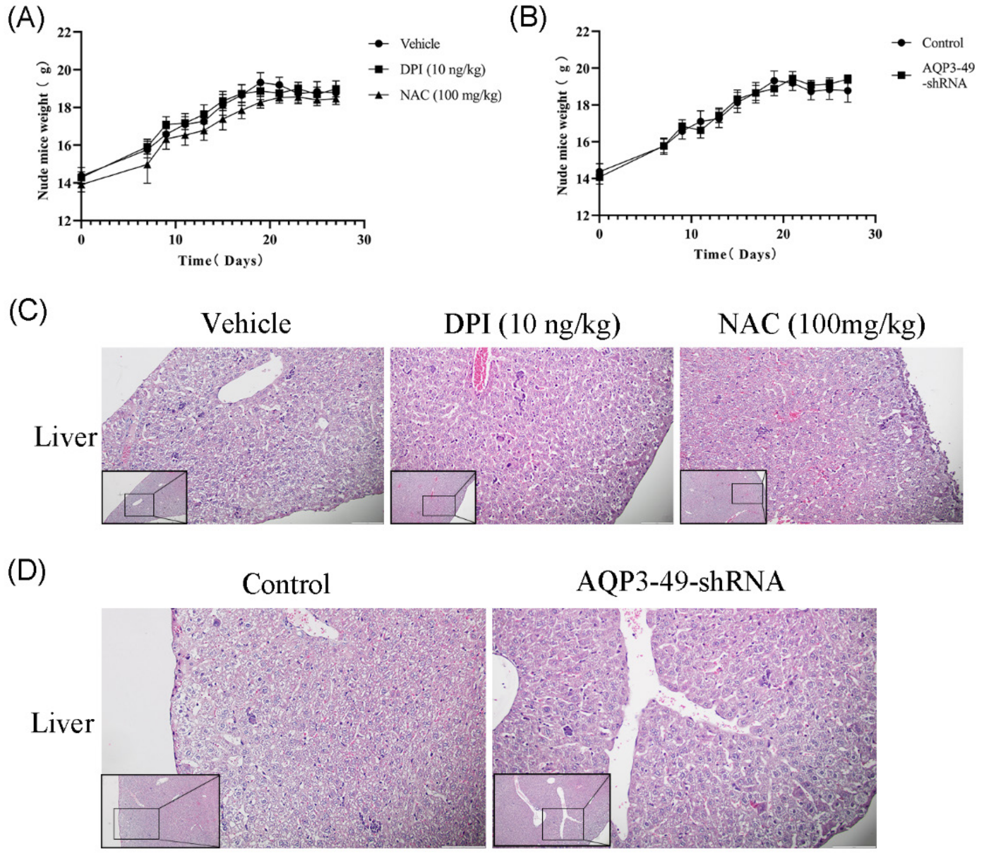

Figure S4. Body weight changes and metastasis in nude mice in the tail vein injection group. (A) Changes in body weight of nude mice in the tail vein injection group after treatment with DPI and NAC. (B) Changes in body weight growth in nude mice injected with AQP3 knockdown cells in the tail vein. (C) HE staining analysis of hepatic sinusoidal metastases in the liver after DPI and NAC treatment. (D) HE staining analysis of hepatic sinusoidal metastases in nude mice injected with AQP3 knockdown cells in the tail vein.
